# Cardiovascular Risk in Systemic Inflammatory Arthritis

**DOI:** 10.3390/jcm12082779

**Published:** 2023-04-08

**Authors:** Fabiola Atzeni, Alessandra Alciati

**Affiliations:** 1 Rheumatology Unit, Department of Experimental and Internal Medicine, University of Messina, 98122 Messina, Italy; 2 Department of Clinical Neurosciences, Villa S. Benedetto Menni, 22032 Albese con Cassano, Italy; 3 Humanitas Clinical and Research Center, Rozzano, 20089 Milan, Italy

In recent years, several papers have been published on cardiovascular (CV) involvement, risk, management, and treatment in systemic inflammatory arthritis (SIA), including rheumatoid arthritis, (RA), psoriatic arthritis (PsA), and axial spondyloarthritis (axSpA). An explanation for the increased interest in this topic is that although joint involvement is considered the principal manifestation of SIA, these diseases have also been associated with an enhanced burden of cardiovascular disease (CVD) [1,2,3]. Patients with SIA exhibit a higher CV risk than the general population due to an enhanced prevalence of traditional CV risk factors (hypertension, dyslipidemia, diabetes, obesity, and smoking) and the altered immuno-mediated inflammatory responses related to the diseases themselves [1,2,3,4].

This editorial aims to discuss studies and review articles that have tried to improve our understanding of CV risk and its management in patients with systemic inflammatory athropathies in 2022. First, a review by M. Nurmohamed and his group [5] focused on RA and CV risk factors underlined that both atherosclerosis and RA could be considered chronic, inflammatory diseases, suggesting that the elevated inflammation in RA could explain the higher rate of CV events compared to the general population. Inflammation could explain the excess of CV risk in RA, in association with the classic CV risk factors and metabolic syndrome that have been estimated to account for more than 50% of all CV deaths [5]. Furthermore, reported evidence suggests that the increased CV risk in patients with inflammatory joint diseases is additionally mediated by medications used to treat disease activity, while ethnicity may also play a role [5,6]. Of note, all types of arthritis were associated with an increased risk (on average by 50%) of myocardial infarction (MI), but, in particular, such risk was consistently increased across patients with RA and PsA [5]. Based on this evidence, optimal CV risk assessment and stratification should be essential in RA clinical management. The assessment of CV risk is the first strategy to identify and thus accordingly treat these patients. The estimation of CV risk is performed using conventional algorithms and imaging techniques. However, conventional algorithms (Framingham, SCORE, etc.) underestimate the risk in many RA patients providing a suboptimal risk stratification and consequently limiting proper clinical management, especially in those categorized as having a low to intermediate risk. Several CV imaging techniques have been reported as being useful in assessing CV involvement in RA, both for screening, diagnosis, and follow-up [5]. However, it is still unclear which methods should be used to evaluate the CV risk in SIA patients in clinical practice taking into account costs and availability [6]. Different original studies from numerous groups have supported the review’s recommendations. A Swedish group [7] performed a case–control study (521 pre-symptomatic RA individuals and 1566 controls) in which CVD risk factors (hypertension, elevated ApoB:A1 ratio, BMI, diabetes, smoking) were identified with the Health Surveys of the Medical Biobank, while the comorbidities were determined using the Swedish National Patient Register and Cause of Death Register. The study showed that CVD risk factors observed in pre-RA individuals were associated with future cardiovascular events (CVE) after RA onset even after adjustments for risk factors and treatments. Furthermore, pre-RA individuals had a higher risk of CVE compared with controls. These results support the need for the early assessment of CVD risk in all subjects with pre-clinical RA and RA [7]. A Spanish group [8], in a cross-sectional study including 347 participants (148 RA, 159 SpA, and 40 controls), which measured the carotid intima media thickness (cIMT) and looked for atheromatous plaques using carotid ultrasound, underlined the greater role of disease activity in subclinical atherosclerosis in patients with RA than in those with SpA. Specifically, the authors showed that cIMT correlated with the mean C-reactive protein (CRP) during the previous 5 years in RA but not with CRP levels at the chosen sampling time-point. However, they did not find such differences in patients with spondyloarthritis (SpA). Furthermore, in patients with moderate–high disease activity, cIMT was greater in patients with RA than in those with SpA after adjusting for age, sex, disease course, and cardiovascular risk factors [8]. Finally, subclinical atherosclerosis measured by carotid ultrasound in patients with RA and SpA was comparable when the disease was well controlled. The study suggests the relevance of CRP and inflammation and supports the need for remission achievement in the majority of the patients in order to reduce CV risk.

A study from Poland [9] confirmed that patients with PsA are characterized by a more pronounced proinflammatory and proatherogenic CV risk profile than patients with axPsA and axial SpA. The study showed that in PsA, but not in axSpA, an elevated serum concentration of interleukin-18 (IL-18) was associated with higher disease activity and a proatherogenic lipid profile, thus resulting in a higher CV risk. These results indicate that IL-18 is a critical contributor to these pathological processes and could be a possible future therapeutic target.

Finally, a study evaluated the modifications of cholesterol efflux capacity (CEC) and the atherogenic cholesterol loading capacity (CLC) of serum lipoproteins in patients with AS and PsA before and after treatment with tumor necrosis factor inhibitors (TNFi) and/or methotrexate (MTX). The results demonstrated that in the SpA group, the total and high-density lipoprotein (HDL)–cholesterol increased after treatment, while lipoprotein(a) decreased and CLC was unchanged [10]. Treatment was associated with increased Scavenger Receptor class B type I (SR-BI)-mediated CEC in the AS group. SR-BI- and ABCG1-mediated CEC were negatively associated with inflammatory parameters and positively related to coffee consumption. SR-BI CEC and CLC were positively and negatively associated with endothelial function. The study suggests that anti-rheumatic treatment is associated with favorable modulation of lipoprotein quality and function in SpA, particularly in AS, despite the induced increase in total cholesterol levels. The authors concluded that, if their findings are confirmed in a larger population, this might represent an atheroprotective beneficial effect beyond what is reflected by conventional serum lipid profile [10].

The strengths of these studies include new useful findings for CV management in SIA patients [8] and are of value in clinical practice to help physicians maximize health and quality of life and reduce the CV risk of ASI patients.

The studies have some limitations as they did not examine the impact of comorbidities such as depressive disorders on CV risk [9].

In conclusion, these studies showed that CV involvement might be a pivotal cause of increased mortality in these patients, impacting SIA management and quality of life. Furthermore, the findings can raise doubts about the time of diagnosis and treatment starting (particularly in the era of biological and targeted synthetic DMARDs) since the persistence of inflammation in patients with SIA, or pre-onset SIA increases the likelihood of developing—*-events. Clinicians should also focus on diagnosing CV comorbidity and check for CV risk factors to avoid incorrect management and treatment. However, the available tools and scores can underestimate CV risk in high-risk patients in clinical practice. Further benefits in analyzing the CV impact in SIA patients include identifying a potential easy screening and treatment approach in clinical practice.
